# Supplementary figures and images for: The actin nucleator Spir-1 is a virus restriction factor that promotes innate immune signalling
Source: PLoS Pathog. 2022 Feb 11;18(2):e1010277. doi: 10.1371/journal.ppat.1010277 (PMC8870497; doi:10.1371/journal.ppat.1010277)

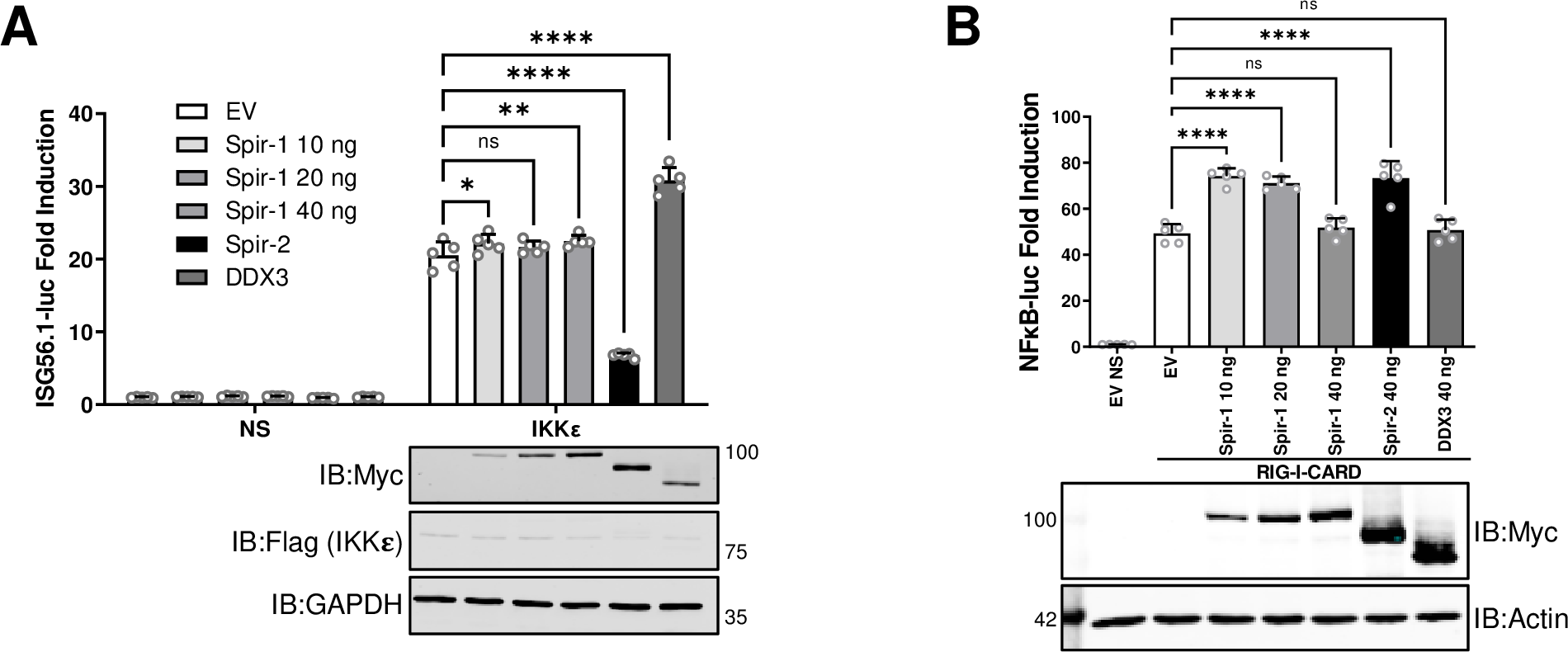

Supplement: S1 Fig — A. Ectopic expression of Spir-1 does not affect IRF3-dependent gene expression induced by IKKε. HEK293T cells was transfected with the ISG56.1 firefly luciferase reporter plasmid, TK-renilla luciferase and plasmids for expression of the indicated proteins. Cells were also co-transfected with EV as the non-stimulated (NS) controls or with the 100 ng of IKKε plasmid to activate the IRF3 pathway. EV was added to samples when necessary to keep the final amount of DNA transfected as 40 ng in all samples. Cell lysates were prepared and analysed as in Fig 2. Data shown are representative of three independent experiments. Immunoblots underneath each graph show the expression levels of the different proteins. The positions of molecular mass markers in kDa are shown on the right and the antibodies used are shown on the left. ns = not significant; *P < 0.05; **P < 0.01, ****P < 0.0001. B. Ectopic expression of Spir-1 affects NF-κB-dependent gene expression induced by the CARD-domain of RIG-I. HEK293T cells was transfected with the NF-κB-firefly luciferase reporter plasmid, TK-renilla luciferase and plasmids for expression of the indicated proteins. Cells were also co-transfected with either EV as the non-stimulated (NS) control or with the 5 ng of the CARD-domain of RIG-I plasmid. EV was added to samples when necessary to keep the final amount of DNA transfected as 40 ng in all samples. Cell lysates were prepared and luciferase expression was measured and normalised to renilla luciferase. Data are expressed as the mean (± SD) fold induction of the firefly luciferase activity normalised to renilla values for the stimulated versus non-stimulated EV sample. Data are representative of three independent experiments and data shown are from at least three individual wells from one representative experiment. Immunoblots underneath each graph show the expression levels of the different proteins. The positions of molecular mass markers in kDa are shown on the left and the antibodies use [file ppat.1010277.s001.tif]
