# Supplementary material for: The actin nucleator Spir-1 is a virus restriction factor that promotes innate immune signalling
Source: PLoS Pathog. 2022 Feb 11;18(2):e1010277. doi: 10.1371/journal.ppat.1010277 (PMC8870497; doi:10.1371/journal.ppat.1010277)
Supplement: S1 Table — List of all plasmids used and their source. (DOCX) [file ppat.1010277.s002.docx]

**S1 Table. Plasmids used in this study.**

| **PLASMID** | **SOURCE** |
| --- | --- |
| human nMyc-Spir-1-pcDNA3.1 | Gift from Prof. Dr. E. Kerkhoff (University Hospital Regensburg, Germany) |
| human nMyc-Spir-2-pcDNA3.1 | Gift from Prof. Dr. E. Kerkhoff (University Hospital Regensburg, Germany) |
| mouse nMyc-B-TrCP-pcDNA3.1 | [1] |
| GFP-Myc-pCMV | [2] |
| nFlag-coK7-pcDNA4/TO | [3] |
| coA49-cFlag-pcDNA3.1 | [2] |
| GFP-cFlag-pcDNA4/TO | Sarah Neidel (Prof. G.L. Smith laboratory) |
| human nMyc-Spir-1-NT-pcDNA3.1 | This study |
| human nMyc-Spir-1-CT-pcDNA3.1 | This study |
| coN1-cTAP-pcDNA4/TO | [4] |
| coC6-cTAP-pcDNA4/TO | [5] |
| nTAP-coN2-pcDNA4/TO | [6] |
| nFlag-coB14-pcDNA4/TO | [3] |
| pcDNA4/TO | Invitrogen |
| nMyc-DDX3-pCMV | Gift from Dr M. Schröder – [7] |
| nHA-DDX3-pCMV | Gift from Dr M. Schröder – [7] |
| IFN-β-luc | Gift from Dr T. Taniguchi (University of Tokyo, Japan) |
| ISRE-luc | Gift from Dr. A. Bowie (Trinity College, Dublin, Ireland) |
| NF-κB-luc | Gift from Dr. A. Bowie (Trinity College, Dublin, Ireland) |
| ISG56.1-luc | Gift from G. Sen (Cleveland Clinic, Cleveland, OH, USA). |
| TK-Renilla | Promega |
| Flag-RIG-I-CARD | Gift from A. Garcia-Sastre (Mount Sinai, New York, USA) |
| HA-MAVS | [5] |
| Flag-TBK1 | [5] |
| Flag-IKKe | [5] |
| IRF3-5D | [5] |
| human nMyc-Spir-1-FFAA-pcDNA3.1 | This study |
| pF3A-nFlag-coK7 | This study |
| pF3A-nMyc-Spir-1 | This study |
| pF3A-nMyc-DDX3 | This study |
| pF3A-F12-cFlag | This study |
| pF3A-nMyc-TAB2 | This study |
| nFlag-coK7-D28A-pcDNA4/TO | This study |
| nFlag-coK7-D31A-pcDNA4/TO | This study |
| nFlag-coK7-DDAA-pcDNA4/TO | This study |
| pSpCas9(BB)-2A-Puro (px459) | [8] - Addgene plasmid #62988 |
| px459-Spir-1-sgRNA#2 | This study |
| pLKO.DCMV.TetO.mcs | [9] |
| pLKO.DCMV.TetO.Myc-Spir-1 | This study |
| pCMV.dR8.91 | Gift from Dr H. Laman [10] |
| pMD-G | Gift from Dr H. Laman [10] |

**References**

[1] Mansur DS, Maluquer de Motes C, Unterholzner L, Sumner RP, Ferguson BJ, Ren H, Strnadova P, Bowie AG, Smith GL (2013) Poxvirus targeting of E3 ligase beta-TrCP by molecular mimicry: a mechanism to inhibit NF-kappaB activation and promote immune evasion and virulence. *PLoS Pathog* 9: e1003183

[2] Neidel S, Ren H, Torres AA, Smith GL (2019) NF-kappaB activation is a turn on for vaccinia virus phosphoprotein A49 to turn off NF-kappaB activation. *Proc Natl Acad Sci U S A* 116: 5699-5704

[3] Torres AA, Albarnaz JD, Bonjardim CA, Smith GL (2016) Multiple Bcl-2 family immunomodulators from vaccinia virus regulate MAPK/AP-1 activation. *J Gen Virol* 97: 2346-2351

[4] Maluquer de Motes C, Cooray S, Ren H, Almeida GM, McGourty K, Bahar MW, Stuart DI, Grimes JM, Graham SC, Smith GL (2011) Inhibition of apoptosis and NF-kappaB activation by vaccinia protein N1 occur via distinct binding surfaces and make different contributions to virulence. *PLoS Pathog* 7: e1002430

[5] Unterholzner L, Sumner RP, Baran M, Ren H, Mansur DS, Bourke NM, Randow F, Smith GL, Bowie AG (2011) Vaccinia virus protein C6 is a virulence factor that binds TBK-1 adaptor proteins and inhibits activation of IRF3 and IRF7. *PLoS Pathog* 7: e1002247

[6] Ferguson BJ, Benfield CTO, Ren H, Lee VH, Frazer GL, Strnadova P, Sumner RP, Smith GL (2013) Vaccinia virus protein N2 is a nuclear IRF3 inhibitor that promotes virulence. *J Gen Virol* 94: 2070-2081

[7] Gu L, Fullam A, Brennan R, Schroder M (2013) Human DEAD box helicase 3 couples IkappaB kinase epsilon to interferon regulatory factor 3 activation. *Mol Cell Biol* 33: 2004-2015

[8] Ran FA, Hsu PD, Wright J, Agarwala V, Scott DA, Zhang F (2013) Genome engineering using the CRISPR-Cas9 system. *Nat Protoc* 8: 2281-2308

[9] Everett RD, Bell AJ, Lu Y, Orr A (2013) The replication defect of ICP0-null mutant herpes simplex virus 1 can be largely complemented by the combined activities of human cytomegalovirus proteins IE1 and pp71. *J Virol* 87: 978-990

[10] Lomonosov M, Meziane el K, Ye H, Nelson DE, Randle SJ, Laman H (2011) Expression of Fbxo7 in haematopoietic progenitor cells cooperates with p53 loss to promote lymphomagenesis. *PLoS One* 6: e21165
