# Supplementary material for: The actin nucleator Spir-1 is a virus restriction factor that promotes innate immune signalling
Source: PLoS Pathog. 2022 Feb 11;18(2):e1010277. doi: 10.1371/journal.ppat.1010277 (PMC8870497; doi:10.1371/journal.ppat.1010277)
Supplement: S2 Table — Sequence information of all primers used. (DOCX) [file ppat.1010277.s003.docx]

**S2 Table. Oligonucleotides used in this study**

| **PLASMID CONSTRUCTION** | **PRIMER** |
| --- | --- |
| human nMyc-Spir-1-CT-pcDNA3.1 | AAAAGCGGCCGCCAGTGTGATGGATGGGTCAGATCTCACTGAT |
|  | AAAAGGATCCGCCGCCGCCATGGAGCAGAAGCTGATCTCCGAGGAGGACCTGGCT |
| human nMyc-Spir-1-NT-pcDNA3.1 | AAAAGCGGCCGCCAGTGTGATGGATGGGTCACTCCTCTGGTGATACAGGCCGCAGCT |
|  | AAGCTTGGTACCGAGCTCGGATCCGCCG |
| pLKO.DCMV.TetO.Myc-Spir-1 | AAAAACGCGTGCCGCCAGTGTGATGGATGGGTCAGATCTCACTGATCGTCCT |
|  | AAAAGCTAGCGCCGCCGCCATGGAGCAGAAGCTGATCTCCGAGGAGGA |
| human nMyc-Spir-1-FFAA-pcDNA3.1 | TTGCCGAACCAGGAGGTTTTCCGCCGCCACTTGGTCTTATACCTGTCAG |
|  | CTGACAGGTATAAGACCAAGTGGCGGCGGAAAACCTCCTGGTTCGGCAA |
| nFlag-coK7-D28A-pcDNA4.1/TO | CGGGACAGCATCATCGCCCTGATCGACGAGTAC |
|  | GTACTCGTCGATCAGGGCGATGATGCTGTCCCG |
| nFlag-coK7-D31A-pcDNA4.1/TO | GCATCATCGACCTGATCGCCGAGTACATCACCTGGCG |
|  | CGCCAGGTGATGTACTCGGCGATCAGGTCGATGATGC |
| nFlag-coK7-DDAA-pcDNA4.1/TO | CGGGACAGCATCATCGCCCTGATCGCCGAGTAC |
|  | GTACTCGGCGATCAGGGCGATGATGCTGTCCCG |
| pF3A-nFlag-coK7 | AAAAGCGATCGCATGGACTACAAAGACGATGACGACAAG |
|  | AAAATTTAAACTCAGTTCAGCTTCTTTTCCAGGAA |
| pF3A-nMyc-Spir-1 | AAAAGCGATCGCATGGAGCAGAAGCTGATCTCCGA |
|  | AAAATTTAAACTCAGATCTCACTGATCGTCCTCTC |
| pF3A-nMyc-DDX3 | AAAAGTTTAAACTCAGTTACCCCACCAGTCAACCC |
|  | AAAAGCGATCGCATGGAGCAGAAGCTGATCTCAGAGGA |
| **CRISPR/Cas9 gRNA targeting region** | **gRNA SEQUENCE** |
| *Spire1* exon 3 | AGAGCAGCTTATCGATCACA |
| **SEQUENCING PRIMERS** |  |
| Forward primer for exon 3 of human *Spire1* | TGGGAAATGTGGAAAATCAACTCG |
| Reverse primer for exon 3 of human *Spire1* | AAAAATAACAGCTTGGACACAGTGG |
