# Supplementary material for: The actin nucleator Spir-1 is a virus restriction factor that promotes innate immune signalling
Source: PLoS Pathog. 2022 Feb 11;18(2):e1010277. doi: 10.1371/journal.ppat.1010277 (PMC8870497; doi:10.1371/journal.ppat.1010277)
Supplement: S3 Table — List of all the primary antibody dilutions used. (DOCX) [file ppat.1010277.s004.docx]

**S3 Table. Primary antibodies used in the study**

| **ANTIBODIES** | **SOURCE** | **DILUTION** |
| --- | --- | --- |
| Rb anti-Myc | Cell Signaling, 2278 | 1 in 1000 |
| Ms anti-Myc | Cell Signaling, 9B11 | 1 in 1000 |
| Rb anti-Actin | Sigma, A2066 | 1 in 1000 |
| Ms anti-Flag | Sigma, F1804 | 1 in 1000 |
| Rb anti-14-3-3 | Santa Cruz, sc-629 | 1 in 1000 |
| Ms anti-Spir-1 | Santa Cruz, sc-517039 | 1 in 500 |
| Ms anti-Spir-1 | Abcam, ab57463 | 1 in 1000 |
| Rb anti-DDX3 | Cell Signaling, 2635 | 1 in 1000 |
| Rb anti-IKKb | Cell Signaling, 2684 | 1 in 1000 |
| Rb anti-HA | Sigma, H6908 | 1 in 1000 |
| Rb anti-C6 | [1] | 1 in 1000 |
| Ms anti-alpha-Tubulin | Millipore, 05-829 | 1 in 5000 |
| Ms anti-GAPDH | Sigma, G8795 | 1 in 1000 |
| Rb anti-IRF3 | Cell Signaling, 4962 | 1 in 1000 |
| Ms anti-COPe | Santa Cruz, sc-133194 | 1 in 500 |
| Rb anti-p-IRF3 | Cell Signaling, 4947S | 1 in 1000 |
| Rb anti IRF3 | Santa Cruz, sc-9082 | 1 in 1000 |
| Rb anti IRF3 | Cell Signaling, 4962 | 1 in 1000 |

**References**

[1] Unterholzner L, Sumner RP, Baran M, Ren H, Mansur DS, Bourke NM, Randow F, Smith GL, Bowie AG (2011) Vaccinia virus protein C6 is a virulence factor that binds TBK-1 adaptor proteins and inhibits activation of IRF3 and IRF7. *PLoS Pathog* 7: e1002247
